# On the growth dynamics of the cyanobacterium *Anabaena* sp. PCC 7938 in Martian regolith

**DOI:** 10.1038/s41526-022-00240-5

**Published:** 2022-10-26

**Authors:** Tiago P. Ramalho, Guillaume Chopin, Lina Salman, Vincent Baumgartner, Christiane Heinicke, Cyprien Verseux

**Affiliations:** Center of Applied Space Technology and Microgravity (ZARM), University of Bremen, 28359 Bremen, Germany

**Keywords:** Biogeochemistry, Biotechnology

## Abstract

The sustainability of crewed infrastructures on Mars will depend on their abilities to produce consumables on site. These abilities may be supported by diazotrophic, rock-leaching cyanobacteria: from resources naturally available on Mars, they could feed downstream biological processes and lead to the production of oxygen, food, fuels, structural materials, pharmaceuticals and more. The relevance of such a system will be dictated largely by the efficiency of regolith utilization by cyanobacteria. We therefore describe the growth dynamics of *Anabaena* sp. PCC 7938 as a function of MGS-1 concentration (a simulant of a widespread type of Martian regolith), of perchlorate concentration, and of their combination. To help devise improvement strategies and predict dynamics in regolith of differing composition, we identify the limiting element in MGS-1 – phosphorus – and its concentration-dependent effect on growth. Finally, we show that, while maintaining cyanobacteria and regolith in a single compartment can make the design of cultivation processes challenging, preventing direct physical contact between cells and grains may reduce growth. Overall, we hope for the knowledge gained here to support both the design of cultivation hardware and the modeling of cyanobacterium growth within.

## Introduction

Space agencies^1^ and private companies^2^ have set goals of sending humans to Mars in the coming decades. In view of recent technological development, and the strong commitment from both public and private actors, this objective now seems realistic. Its achievement may have a tremendous scientific impact: even modest mission architectures, with a small crew spending less than two years on the surface, could lead to scientific discoveries which are staggering by their number and their importance. Those, however, would only be a small sample of what could be learned with a permanent research station, or perhaps slightly larger settlements built around science and exploration.

Long-term crewed infrastructures should be as independent of Earth as possible in terms of material resources: the price per kilogram of an imported payload, as well as the uncertain success of resupply missions, would otherwise bring costs, risks, and logistical difficulties to unsustainable levels. This independence may be reached with the help of biological systems. On Earth, we rely heavily on them for a wide range of compounds including food items, fermented drinks, pharmaceuticals, structural materials, and fuels. They may become even more central on Mars^3–6^: constraints are such that bioprocesses would often be more resource-efficient than abiotic industries used for similar purposes on Earth.

**Fig. 1:**
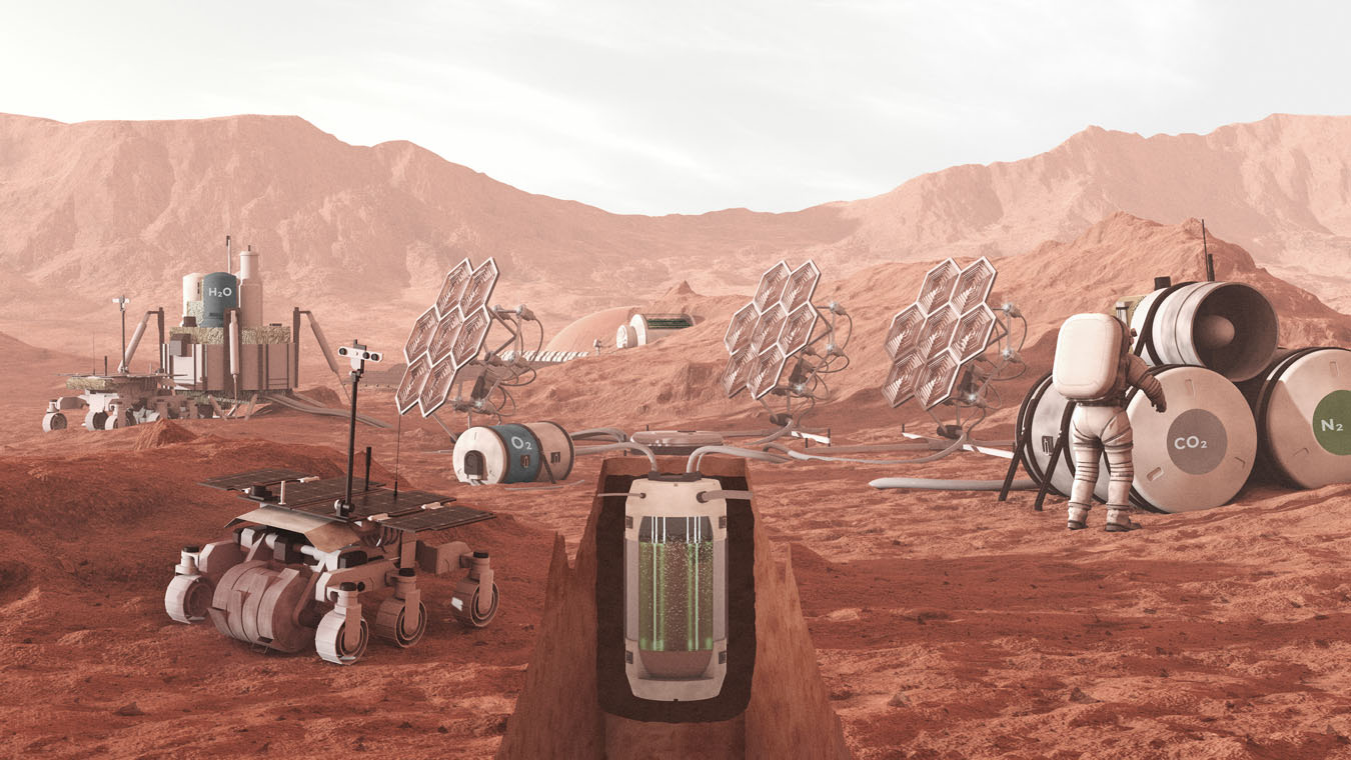
Artistic representation of a system where diazotrophic, rock-leaching cyanobacteria are grown using materials available on Mars. In that system, the necessary water is mined from the ground^49^; carbon and nitrogen are sourced from the atmosphere and provided at a low (though higher than Mars-ambient) pressure^7^; and mineral nutrients are obtained from the regolith^7,8,10,50^. Lighting is provided as collected sunlight supplemented, when needed, with LEDs. Cyanobacteria could produce consumables such as O_2_ and dietary proteins, but also support the growth of secondary producers^9^ – which, in turn, could generate products ranging from food to materials to pharmaceuticals to fuels. Here, cyanobacteria are depicted in a photobioreactor containing regolith, deposited at the bottom, and a liquid phase stirred by bubbling. The photobioreactor is buried to protect cultures, hardware and operators against dust and radiation, as well as to improve thermal stability. The mining of water is represented with an extraction plant on the left, and that of regolith with excavation rovers. A gas separation and compression module, on the right, symbolizes the provision of gases from the Martian atmosphere. Lighting is shown as Fresnel lenses and light guides. Cultivation products are represented by an oxygen storage tank and a greenhouse in the background. Artwork: Joris Wegner, University of the Arts Bremen.

On the other hand, biological systems must be provided with suitable nutrients. While a limited amount of imported feedstock could be recycled, recycling alone would mean that the amounts of available resources only decrease over time. This could be solved with the sustainable use of local resources (in-situ resource utilization; ISRU). Although most organisms cannot utilize raw Martian resources directly, it has been argued that some cyanobacteria – notably some members of the *Nostocaceae* family – could be grown from materials available in the local ground and atmosphere. They could then be used as feedstock for other organisms of biotechnological interest^7–10^, thereby setting the ground for ISRU-based, sustainable bioprocesses on Mars (Fig. 1). Efforts aimed at developing that concept have recently intensified^7,11–16^; evidence that suggests its feasibility accumulates. But feasibility does not suffice to decide whether a system should be integrated into mission plans: its cost-efficiency must be determined and compared to potential alternatives. This is hindered by a lack of knowledge on how the factors which are limiting in ISRU-based cultivation affect cyanobacterium physiology.

One of those factors pertains to the partial pressure of dinitrogen: a tradeoff is required between maintaining a low total pressure (which would ease constraints on engineering) and providing abundant nitrogen^7^. Another is the suitability of Mars’s regolith as feedstock, since it is the expected source of most nutrients. A third factor is the toxicity of perchlorates (compounds thought to be ubiquitous on the Martian surface^17,18^): unless remediation is performed, using regolith as a nutrient source means introducing perchlorates into the cultivation medium.

The first factor is the object of another line of work^7^. Here we focus on the latter two, the suitability of regolith as feedstock and the toxicity of perchlorates, which we examine together as their cases are tightly intertwined. The composition of the Martian regolith (which is mostly basaltic) is known from Martian meteorites on Earth, Mars-orbiting spacecraft, landers and rovers^19–24^. Various *Nostocaceae* can use minerals of similar composition as nutrient sources^7,25,26^. However, little information is available on the concentration-dependent growth dynamics in such regolith, on what mechanisms constrain those dynamics, or on how growth could be enhanced. Even less can be predicted for regolith that contains perchlorates: increasing substrate concentrations implies increasing both nutrients and toxins, the effects of which may not be independent.

We address these knowledge gaps in the present work. *Anabaena* sp. PCC 7938 was chosen as a model strain, based on previous assessments of its ability to utilize resources available on Mars and of its suitability as feedstock for other organisms^16^. We determine its growth dynamics as a function of concentrations of a Martian regolith simulant (used as the source of mineral nutrients), of perchlorates, and of a combination of both. To help devise growth improvement strategies and predict the effects of regolith that differs from the simulant used here, we identify the limiting nutrient and determine growth dynamics as a function of its concentration. In both cases, growth dynamics are fitted to mathematical models so the results can be extrapolated to other scenarios. We also investigate whether physically separating cyanobacteria and regolith (to facilitate the provision of adequate lighting, as well as various processes associated with cultivation) would affect cell-mineral interactions.

Overall, our results should help assess the cost-efficiency of ISRU-based bioprocesses on Mars that rely on cyanobacteria – and determine the mission architectures for which they would be suitable solutions.

## Results

### Growth dynamics of *Anabaena* sp. PCC 7938 in perchlorate-free MGS-1

**Fig. 2:**
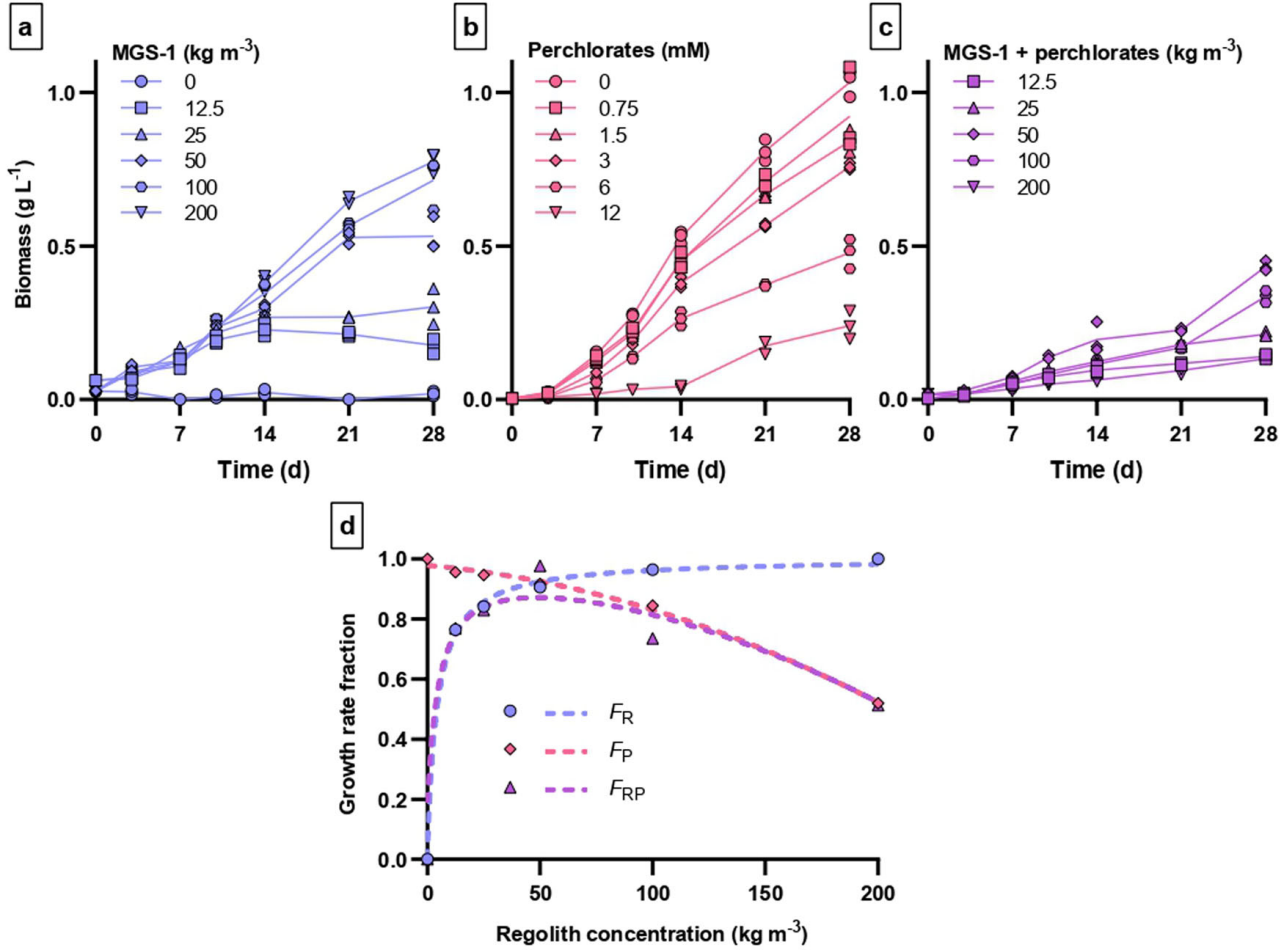
Growth of *Anabaena* sp. PCC 7938 with regolith and perchlorates. **a** Growth curves in bi-distilled and de-ionized water (ddH_2_O) supplemented with various concentrations of a perchlorate-free simulant of Martian regolith (MGS-1). **b** Growth in BG11_0_ spiked with various concentrations of perchlorate ions. **c** Growth in ddH_2_O supplemented with various concentrations of MGS-1, and spiked with perchlorate ions at concentrations corresponding to 0.6 wt% of the indicated regolith concentrations. **d** Normalized growth rate as a function of regolith concentration (*F*_R_), perchlorate concentration (*F*_P_), or concentration of perchlorate-containing regolith (*F*_RP_). Symbols correspond to experimental data. Lines represent equations fitted to the corresponding experimental results (*F*_P_, *F*_R_) or the product of these equations (*F*_RP_ = *F*_P_ × *F*_R_).

*Anabaena* sp. PCC 7938 was cultivated in water supplemented with the MGS-1 Mars Global Simulant^27^ (MGS-1*)*, at concentrations ranging from 12.5 to 200 kg m^−3^, to determine its regolith-dependent growth dynamics in the absence of perchlorates. Growth curves are given in Fig. 2a. Results show that growth rates can be predicted with a Monod equation^28^ (*R*^2^ = 0.998) with a half-velocity constant (*K*_R_) of 4.247 kg m^−3^ and a maximum growth rate (*µ*_maxR_) of 2.177 ×10^−6^ s^−1^ (Fig. 2d). Though growth rates increased only slowly with regolith concentrations above ca. 50 kg m^−3^, the final biomass concentrations varied substantially throughout the tested range.

### Growth dynamics of *Anabaena* sp. PCC 7938 in presence of calcium perchlorate

In order to determine the concentration-dependent effects of perchlorate ions on growth kinetics, *Anabaena* sp. PCC 7938 was cultivated in BG11_0_ spiked with various concentrations of calcium perchlorate. Concentrations were selected to cover the range of perchlorate ions that would be obtained if adding, to water, 0 to 200 kg m^−3^ of regolith containing 0.6 wt% of perchlorate ions. Growth curves are given in Fig. 2b. The resulting growth dynamics can be derived into a second-order polynomial equation (*R*^2^ = 0.994; Fig. 2d):

1 $${{{\mathrm{\mu }}}}_{{{\mathrm{P}}}} = {{{\mathrm{\mu }}}}_{{{{\mathrm{Pmax}}}}}-K_{{{{\mathrm{P}}}}1} \times C_{{{{\mathrm{ClO}}}}4 - }^2 - K_{{{{\mathrm{P}}}}2} \times {{{\mathrm{C}}}}_{{{{\mathrm{ClO}}}}4 - }$$

In that equation, *μ*_P_ is the specific growth rate at a given perchlorate concentration $(C_{{\mathrm{ClO4}} - })$, *μ*_Pmax_ is the maximum growth rate (5.042 × 10^−6^ s^−1^), and *K*_P1_ and *K*_P2_ correspond to perchlorate inhibition constants (1.12 × 10^−8^ and 5.70 × 10^−8^, respectively).

### Combined effect of MGS-1 and perchlorate on the growth of *Anabaena* sp. PCC 7938

After estimating the dose-dependent effects on growth dynamics of MGS-1 and perchlorate separately, we sought to determine whether those effects were independent, or interacting, when combined. We assumed independence: that the fraction of the maximum growth rate obtained as a function of perchlorate-containing regolith (*F*_RP_) could be predicted by multiplying the fractions obtained as a function of regolith concentration (*F*_R_) and of perchlorate concentration (*F*_P_) separately. This was tested by cultivating *Anabaena* sp. PCC 7938 in water supplemented with MGS-1 (at concentrations ranging from 0 to 200 kg m^−3^) and spiked with calcium perchlorate. Concentrations of both compounds were matched so that perchlorate ion concentrations corresponded to 0.6 wt%. of MGS-1 concentrations. Growth rates increased with regolith concentrations up to ca. 50 kg m^−3^, then decreased (Fig. 2c, d). The experimental data fitted the curve generated by assuming multiplicative kinetics (*F*_R_ × *F*_P_ = *F*_RP_; Kolmogorov-Smirnov test, *P* > 0.98).

### Limiting nutrient, and effects on growth rates of changes in its concentration

**Fig. 3:**
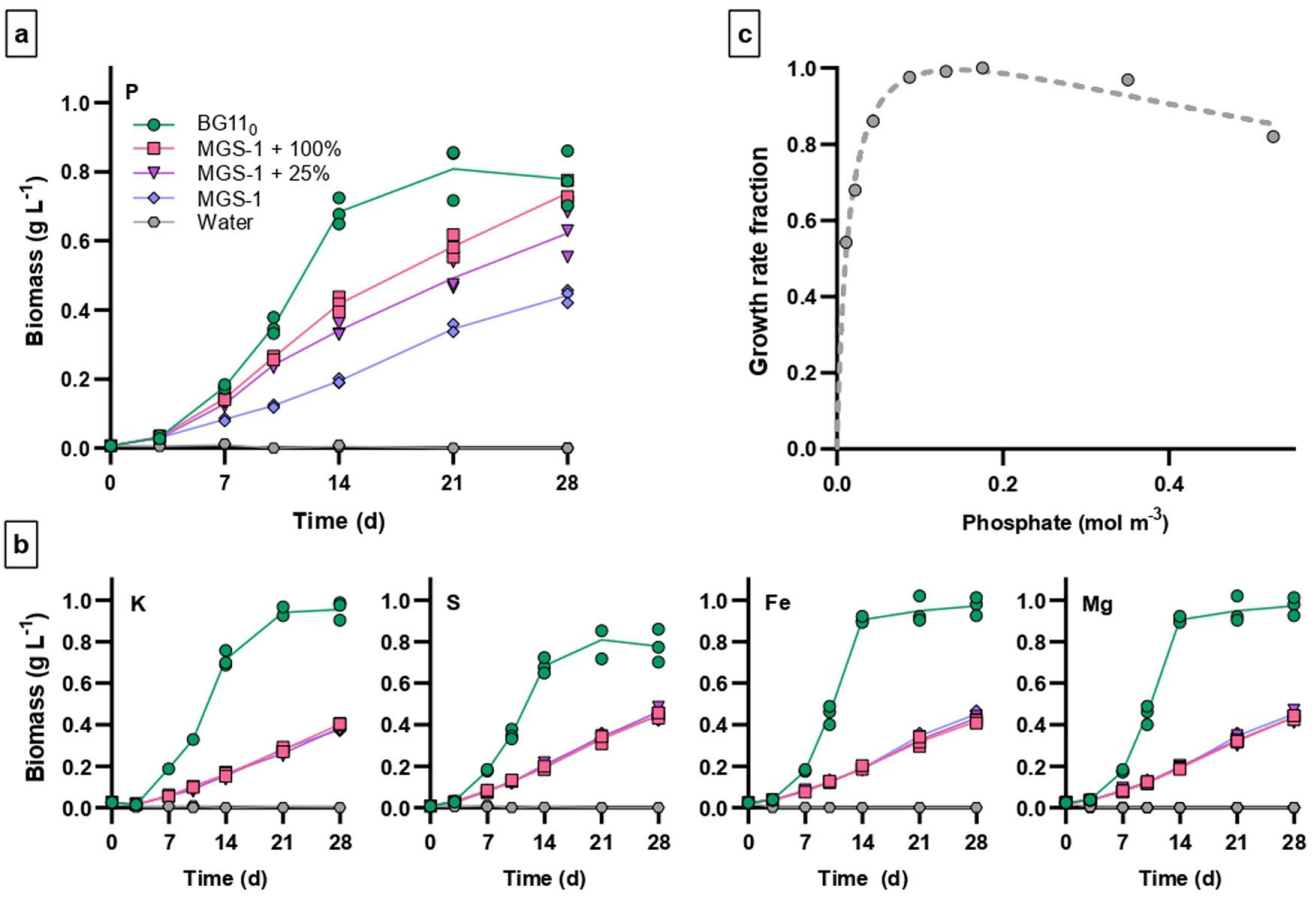
Identification of the element which is limiting when *Anabaena* sp. PCC 7938 utilizes MGS-1 as a nutrient source. **a**, **b** Growth curves in BG11_0_ (circles), in ddH_2_O (hexagons), or in ddH_2_O containing MGS-1, unsupplemented (diamonds) or supplemented with phosphorus (Na_2_HPO_4_; P); potassium (KCl; K); sulfur (Na_2_SO_4_; S); iron (FeCl_3_; Fe) or magnesium (MgCl; Mg) at concentrations corresponding to those found in BG11_0_ (squares) or a quarter of them (triangles). **c** Normalized growth rate in BG11_0_ as a function of the concentration of phosphate (provided as Na_2_HPO_4_).

To further characterize and predict regolith-dependent growth, we sought to identify the limiting nutrient (i.e., the nutrient whose scarcity, relative to cyanobacterial needs, limits growth) and determine the associated growth dynamics. *Anabaena* sp. PCC 7938 was therefore cultivated in water containing MGS-1 and supplemented with one of the five elements not provided by water or atmospheric gases which are most represented in the biomass (K, S, P, Mg, and Fe). Only phosphorus improved growth (Fig. 3a, b). Adding phosphorus at the concentration found in BG11_0_ or a quarter of it increased biomass after 28 days by 67% or 41%, respectively. As this identified phosphorus as the limiting nutrient, we characterized its concentration-dependent effect on growth rates by cultivating *Anabaena* sp. PCC 7938 in increasing phosphate concentrations. Growth fractions matched a Haldane equation^29^ (*R*^2^ = 0.973) with a half-velocity constant for phosphate (*K*_PHOS_) of 0.0149 mM and an inhibition constant (*K*_iPHOS_) of 1.379 mM (Fig. 3c).

### Shading effect of resuspended MGS-1

**Fig. 4:**
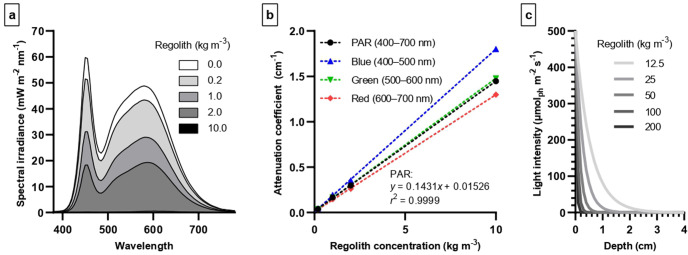
Shading effect of suspended MGS-1. **a** Spectral irradiance measured underneath 3.3 cm of water, or of water containing MGS-1 (grain size <100 µm) at the indicated concentrations. **b** Attenuation coefficients of a suspension of MGS-1 in water, as a function of regolith concentration and for different spectral ranges (calculated from spectral irradiance measurements). **c** Light intensity as a function of depth, for different concentrations of suspended regolith, assuming an intensity of 500 μmol_ph_ m^−2^ s^−1^ at the surface (calculated from the PAR attenuation coefficient).

A homogeneous cultivation volume, where regolith grains are suspended, may be considered in a photobioreactor for the optimization of gas exchange and nutrient availability, as well as to facilitate the in- and outflow of substrates and products. However, shading by regolith grains would increase the difficulty and cost of optimizing light conditions. In order to assess shading intensity, we determined the effect of regolith suspensions on spectral irradiance in the photosynthetically active radiation (PAR) range. Even with a short light path (3.3 cm), regolith concentrations an order of magnitude below otherwise optimal values reduced irradiance dramatically (Fig. 4a), bringing it below detection levels at 20 kg m^−3^. Absorption coefficients in the PAR, red, green and blue ranges are given in Fig. 4b; absorption was most intense in the blue and least in the red. An estimate of the light intensity as a function of depth, for different concentrations of suspended regolith and assuming a surface intensity of 500 μmol_ph_ m^−2^ s^−1^, is given in Fig. 4c.

### MGS-1-dependent growth of *Anabaena* sp. PCC 7938 without direct cell-regolith contact

**Fig. 5:**
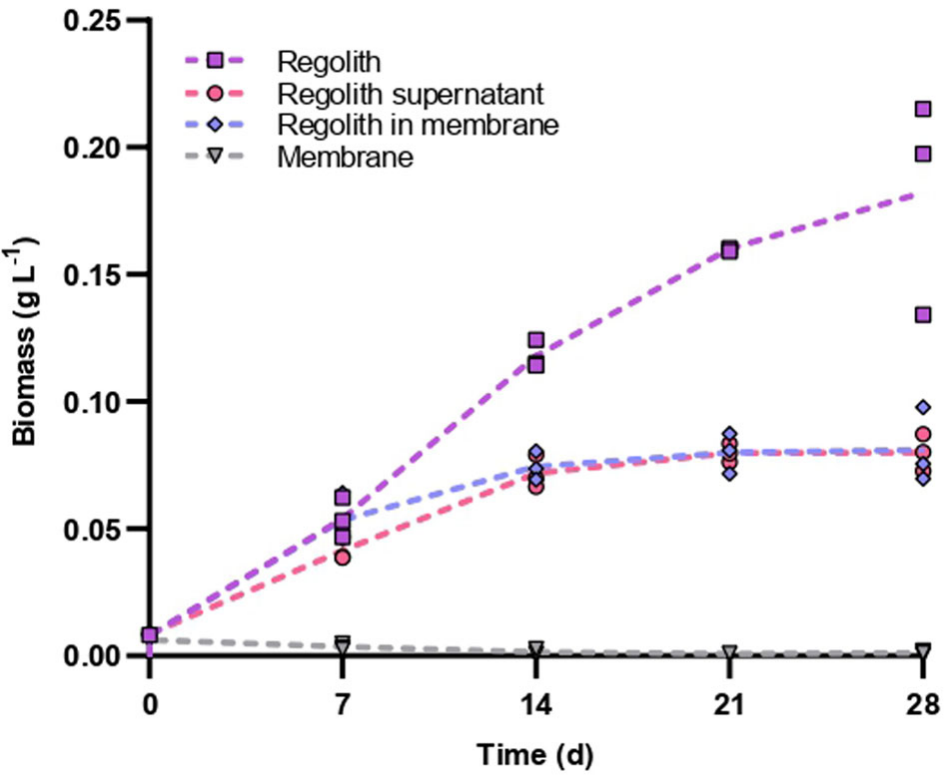
Growth of *Anabaena* sp. PCC 7938 when utilizing MGS-1, with or without direct cell-grain contact. Cyanobacteria were grown in ddH_2_O containing 200 kg m^−3^ of MGS-1 (regolith; squares), ddH_2_O in which MGS-1 had been incubated for 28 days and from which it had been removed (regolith supernatant; circles), ddH_2_O with 200 kg m^−3^ of MGS-1 contained in a cellulose hydrate dialysis membrane (regolith in membrane; diamonds), or ddH_2_O with an empty dialysis membrane (membrane; triangles). Cultures were shaken but in such a way that MGS-1 would remain at the bottom of the flasks, thereby minimizing cell shading.

Given the high shading effect of regolith, photobioreactor designs may be considered where cells and regolith grains are maintained in connected (for soluble molecules), but physically separated, compartments. However, the lack of direct contact may limit the capability of cyanobacteria to utilize regolith as a nutrient source. We thus determined, first, whether cyanobacteria had an effect on nutrient release from the regolith. This was done by comparing the growth of cyanobacteria in water containing regolith and in water where regolith had been incubated (with conditions and duration equivalent to those of cultivation), then removed. Growth was higher when cells and regolith were incubated together (Fig. 5): biomass was more than twice the amount after 28 days (0.08 ± 0.01 vs 0.18 ± 0.04 g L^−1^), suggesting a positive effect of the cyanobacteria on nutrient release. We then tested whether this putative effect could be obtained, at least in part, in a system where cells and regolith were separated with a dialysis membrane that prevented direct cell-grain contact but allowed for the diffusion of molecules smaller than 15 kDa. Results suggest otherwise: growth matched that observed when cells and regolith grains were incubated one after the other (Fig. 5).

## Discussion

Diazotrophic cyanobacteria may enable the utilization of resources naturally available on Mars as feedstock for bioprocesses, thereby supporting the sustainability of future missions to the red planet. Efficiency will be determined to a large extent by the dynamics of cyanobacterium growth when relying on regolith as a nutrient source; characterizing those dynamics is therefore critical to an estimation of the concept’s relevance. The work presented here is a step in that direction. We first described the growth dynamics of the cyanobacterium *Anabaena* sp. PCC 7938 in presence of MGS-1, a simulant of the Rocknest windblown soil at Gale crater (Mars). We identified phosphorus as the limiting nutrient in MGS-1 and consequently determined the impact of phosphate concentrations on the cyanobacterium’s growth dynamics. We described the concentration-dependent effects of perchlorates on growth rates and determined whether these effects were interacting with those of using regolith as a nutrient source, which we did not find to be the case. Finally, we gathered results which suggest that, while maintaining regolith in suspension would hinder the provision of adequate lighting, preventing a direct physical contact between cells and regolith grains would affect the mobilization of nutrients. Taken together, the insights obtained here can support both the design of ISRU cultivation hardware and the modeling of cyanobacterium growth within.

MGS-1 had previously been shown to support the growth of *Anabaena* sp. PCC 7938^7^; here we determined its concentration-dependent effect on growth rates, which we found to fit a Monod equation. This equation can support rough assessments of cyanobacterium productivity when relying on Martian regolith as a nutrient source. However, differences exist between MGS-1 and the soil it simulates. Besides, the regolith is not homogeneous at a planetary scale: it may vary, for instance, in grain size and mineralogical composition. In order to support predictions of growth dynamics with regolith differing slightly from MGS-1, we sought to identify the limiting element. It appeared to be phosphorus. That is not unexpected: this element plays a central role in biochemistry and represents large fractions of molecules as essential as nucleic acids, ATP and phospholipids^30^. The growth of primary producers (including cyanobacteria) in natural ecosystems is commonly dictated by the availability of phosphorus and nitrogen^31^, and *Anabaena* sp. PCC 7938 can utilize the latter as N_2_ (which is usually not limiting under ambient partial pressure^32^). It is noteworthy that MGS-1 is poorer in phosphorus than the regolith unit it is based on (ca. 0.4 wt% P_2_O_5_ equivalent in MGS-1^27^ vs 1 wt% at Rocknest^33^) and that, more broadly, Mars is richer in phosphorus than Earth (see for instance Table S1 in Adcock et al., 2013^34^). In addition, the dominant phosphate-bearing minerals on Mars (chlorapatite, whitlockite and merrillite) tend to have larger phosphorus release rates during dissolution in water than their terrestrial counterpart (fluorapatite)^34^. The availability of phosphorus is thus expected to be much higher when using actual Martian regolith.

We consequently provide a description of phosphate’s concentration-dependent effects, which can help assess growth under release rates differing from that of MGS-1. However, although cyanobacteria grown in MGS-1 reached the same biomass after 28 days as in BG11_0_ when phosphate concentrations were matched, growth rates were lower and that biomass was reached later. This may be due to the release rates of another mineral becoming limiting. A better understanding of the processes associated with regolith dissolution in water, as well as the development of simulants whose composition reflect more accurately that of the simulated regolith, could help identify the most suitable regolith units on Mars. Different units could also be combined to improve the overall composition, and the average grain size could be reduced to increase surface area (and therefore the extent and rate of nutrient release) per mass unit. If considering the regolith as a nutrient source only, our assessment of its concentration-dependent effects on growth rates thus seems conservative.

But Mars’s regolith is not a source of nutrients only: critically, it also contains oxychlorine species. Most concerning are likely perchlorates: they are toxic to cyanobacteria, with intensities which are species-dependent^14,35^. Here, spiking MGS-1 with 0.6 wt%. of perchlorate ions (a seemingly typical mass fraction on Mars^36^) reduced the growth rates of *Anabaena* sp. PCC 7938 in 200 kg m^−3^ of MGS-1 by approximately a half. The optimum MGS-1 concentration was shifted from 200 kg m^−3^ to ca. 50 kg m^−3^, above which increases in perchlorate toxicity seemed to counteract the benefits of a higher nutrient release. We used calcium perchlorate as the source of perchlorate ions, as it is presumed to be the dominant parent salt at Rocknest^37^; but perchlorate salts dissolve readily in water and we do not expect the parent salt to have a large effect on the toxicity of the perchlorate ions. On the other hand, concentrations vary with location, and how concentration changes with depth in the regolith is not known^38^. Besides, the regolith (or the water to which it has been added) could be processed to decrease perchlorate concentrations; a variety of methods have been developed on Earth^39^, where perchlorates are widespread contaminants. Cyanobacteria could also be engineered for increased perchlorate resistance. We wondered whether growth in regolith with different perchlorate mass fractions could be predicted from the respective concentration-dependent effects of perchlorates as a toxin and of a dependence on regolith as a nutrient source: stress factors often have synergistic effects^40^. This was not found to be the case here. Results rather suggest multiplicative kinetics, although our sample size was too low to exclude with certainty a small deviation from it. The equations we provide can thus be used for rough estimates of cyanobacterial growth rates in regolith with other perchlorate mass fractions.

Our description of *Anabaena* sp. PCC 7938’s growth dynamics in MGS-1 does not shed light on the mechanisms by which nutrients are mobilized. When microorganisms rely on rock weathering, nutrient availability depends upon rock dissolution, which itself depends on a complex (and only superficially understood) set of interrelated factors such as primary mineral composition, microbe-substrate interactions, and the formation of secondary minerals^41–43^. In the absence of biological systems, various components from basaltic substrates can dissolve in water and release mineral nutrients^44^; but that alone is not responsible for the growth documented here, as confirmed by the assays where we used water in which regolith had been incubated before removing undissolved grains. Consistently, several *Nostocaceae* species were shown to intensify the release of ions from rocks^8,42^. What mechanisms are involved for those species specifically is mostly unknown. On the other hand, contributing mechanisms have been identified in various other organisms: they include pH alteration, shifts in concentrations of dissolved minerals away from equilibrium (via the consumption of nutrients, or by fostering precipitation), enzyme-catalyzed oxidation or reduction, and the secretion of ligands, organic acids, and other substances that accelerate breakdown^45,46^. Some require a direct contact between cells and minerals; others depend on soluble molecules^47^. An example of the latter case which is thought to be dominant in some cyanobacteria is a change in pH^42^: cyanobacterial metabolism tends to alkalinize media and pH impacts basalt dissolution rates.

Knowing the relative importance of contactless mechanisms is critical to the modeling and optimization of ISRU-based growth. Indeed, regolith grains pose challenges to a photobioreactor’s design and operations. First, it is hard to separate cells from minerals when both have been mixed: grains can be as small as cells and settle down as slowly, limiting the applicability of centrifugation or filtration. Second, regolith in suspension creates strong shading: based on our measurements of MGS-1 in water, white light at a surface intensity of 500 μmol_ph_ m^−2^ s^−1^ would be attenuated to below 1 μmol_ph_ m^−2^ s^−1^ by 12.5 kg m^−3^ of regolith within a depth of 35 mm, or by 200 kg m^−3^ within 2 mm. In the cultivation assays described here, cultures were either static or gently stirred, allowing MGS-1 to settle down. Cultures in a photobioreactor, by contrast, are typically stirred vigorously to maintain uniform conditions (e.g., in terms of temperature, pH, and nutrient concentrations), enhance gas exchange, and reduce sedimentation, clumping, and fouling. This would resuspend the regolith and prevent adequate lighting. But if cyanobacterium-mineral interactions can occur for the most part without direct contact, cells and regolith might best be maintained in connected (for soluble molecules), but physically separate, compartments.

We thus tested whether regolith grains could be separated from cells by a dialysis membrane, which would prevent direct cell-grain contact but allow for the exchange of small molecules. This setup presumably did not alter the effects on regolith dissolution of cyanobacterium-mediated pH changes, or of a shift in equilibrium mediated by the consumption of released ions. Growth, however, was reduced sharply by this segregation: it was similar to that observed when cells and regolith were incubated sequentially. This suggests that a direct contact between cells and grains is required for *Anabaena* sp. PCC 7938 to foster the dissolution of regolith grains, or at least the transfer of molecules larger than the cutoff of the dialysis membrane (15 kDa). In either case, the design of dedicated hardware and processes is required to optimize both the cultivation of cyanobacteria in regolith and the subsequent purification of the biomass.

The work presented here does not address all aspects of cyanobacterium cultivation from local resources: elements not pertaining directly to the regolith, such as the provision of gases from the Martian atmosphere, the control of temperature or the optimization of the light spectra, require similar attention. However, the utilization of regolith by cyanobacteria is one of the most critical and among the least known; our results should thus contribute significantly to the modeling and optimization of ISRU-based cyanobacterium cultivation systems. Those systems, in turn, may support the sustainability of future missions to Mars.

## Methods

### Strain and routine growth conditions

*Anabaena* sp. PCC 7938 was obtained from the Pasteur Culture Collection of Cyanobacteria (Paris, France). Cultures were maintained inside a poly klima PK 520-LED photo-incubator at 25 °C, in BG11_0_ medium, with a light intensity of 5–10 μmol_ph_ m^−2^ s^−1^ (16 h/8 h day/night cycle). Prior to experiments, cultures to be used for inoculation were cultivated on a rotary shaker at 100 rpm under a light intensity of 15–20 μmol_ph_ m^−2^ s^−1^.

### Growth dynamics in perchlorate-free MGS-1

MGS-1 was purchased from Exolith Lab (Orlando, Florida, USA; shipped May 2019). Its concentration-dependent effects on PCC 7938 growth dynamics were determined by cultivating cells in double-distilled and deionized water (ddH_2_O) supplemented with MGS-1 at 0, 12.5, 25, 50, 100, and 200 kg m^−3^. This was performed in 24-well plates, in triplicate for each time point. The inoculum was prepared by washing a preculture twice in ddH_2_O and delivered to an optical density at 750 nm of 0.02. The inoculated plates were sealed with parafilm (to reduce evaporation), in which one longitudinal slit of ca. 2 cm was cut on each side to enhance gas exchange. Plates were incubated for 28 days at 25 °C, under ambient air, with a light intensity of 15–20 μmol_ph_ m^−2^ s^−1^ (16 h/8 h day/night cycle) and, to avoid shading from suspended regolith grains, without shaking. After 0, 3, 7, 10, 14, 21, and 28 days, chlorophyll a was extracted with ethanol from the whole of each sample and quantified based on optical density at 665 nm^48^. The final biomass in each sample was inferred from chlorophyll a concentrations, using a conversion factor determined experimentally^16^. Growth rates in the active growth phase were calculated, normalized to the highest value for MGS-1 samples (obtained at 200 kg m^−3^; preliminary results showed that increasing concentrations past this did not increase growth) and fitted to a Monod equation^28^ using the non-linear curve fitting function of GraphPad Prism.

### Growth dynamics in the presence of perchlorates

In order to determine whether the dose-dependent effects on growth dynamics of MGS-1 and perchlorate were independent, PCC 7938 was cultivated in BG11_0_, in BG11_0_ spiked with calcium perchlorate (Sigma-Aldrich, Merck), or in ddH_2_O supplemented with MGS-1 (at 12.5 and 25, 50, 100, and 200 kg m^−3^) and spiked with perchlorate. Concentrations of both compounds were matched so that perchlorate ion concentrations corresponded to 0.6 wt% of MGS-1 concentrations. Growth curves of PCC 7938 in BG11_0_ spiked with perchlorate concentrations corresponding to 0.6% of 50, 100 and 200 kg m^−3^ (but without MGS-1) were already available^16^ and were here used again. Cultivation was prepared and performed, and biomass determined and compared, as described above (section “Growth dynamics in perchlorate-free MGS-1”). Growth rates in the active growth phase were calculated and normalized to those in perchlorate-free ddH_2_O supplemented with 200 kg m^−3^ of MGS-1 (for samples containing MGS-1) or in perchlorate-free BG11_0_. The resulting fractions were fitted to a second-degree polynomial equation using the curve-fitting feature of GraphPad Prism. A Kolmogorov-Smirnov test was applied to determine how well the expected relationship between growth rates and concentrations of perchlorate-containing regolith, predicted assuming multiplicative kinetics and based on the effects of MGS-1 and perchlorates separately (i.e., assuming *F*_R_ × *F*_P_ = *F*_RP_), fitted experimental results.

### Identification of the nutrient limiting regolith-based growth

The limiting nutrient, when *Anabaena* sp. PCC 7938 feeds on MGS-1, was identified as follows. Cells were grown in ddH_2_O containing 200 kg m^−3^ of MGS-1 supplemented with phosphorus (Na_2_HPO_4_; 0.1753 and 0.0438 mM); potassium (KCl; 0.3501 and 0.0875 mM); sulfur (Na_2_SO_4_; 0.3041 and 0.0760 mM); magnesium (MgCl; 0.3045 and 0.0761 mM); or iron (FeCl_3_; 0.0226 and 0.0056 mM). The concentrations selected for each element correspond to 100 and 25% of those at which they are found in standard BG11_0_. Additional samples contained unsupplemented MGS-1 in ddH_2_O; regolith-free BG11_0_; and regolith-free ddH_2_O. Cultivation was prepared and performed, and biomass determined and compared, as described above (section “Growth dynamics in perchlorate-free MGS-1”). For each time point, the biomass in each supplemented sample was compared to that in ddH_2_O containing unsupplemented MGS-1.

### Determination of the half-velocity and inhibition constants for phosphate

After phosphorus availability was identified as limiting when PCC 7938 relies on MGS-1, the Haldane half-velocity and inhibition constants for phosphate were determined. PCC 7938 was grown in a medium supplemented with Na_2_HPO_4_ in a range of concentrations (0.011, 0.022, 0.044, 0.088, 0.131, 0.175, 0.350, and 0.525 mM). The base medium was a modified BG11_0_, prepared using ddH_2_O as a solvent and where the KH_2_PO_4_ salt was substituted with KCl (0.3501 mM) so the concentrations of phosphorus and potassium would be independent. ddH_2_O and standard BG11_0_ were added as controls. Cultivation was prepared and performed, and biomass determined and compared, as described above (section “Growth dynamics in perchlorate-free MGS-1”). Growth rates in the active growth phase were calculated, normalized to that in standard BG11_0_ and fitted to a Haldane equation for substrate inhibition^29^ using the non-linear curve fitting function of GraphPad Prism.

### Shading by suspended MGS-1

A rough estimate of the shading which would be caused by Martian regolith, if in suspension, was obtained by determining the light attenuation coefficient of suspended MGS-1. Seven glass beakers were filled with dH_2_O to a height of 3.3 cm. MGS-1 was sieved to exclude grains larger than 100 μm (to allow for its full suspension) and added to six of the beakers, to concentrations of 0.1, 0.2, 1, 2, 10, or 20 kg m^−3^. Samples were placed under a white light, and the spectral irradiance was measured underneath the beakers with a Mavospec Base spectrometer (Gossen). Irradiance underneath the most concentrated sample was below detection; this sample was not considered further. The attenuation coefficient of MGS-1 in the PAR range (400–700 nm), as well as in the blue (400–500 nm), green (500–600 nm), and red (600–700 nm), were calculated from irradiance data using the Lambert-Beer law. Light intensity as a function of depth was calculated using the PAR attenuation coefficient.

### Growth in MGS-1 without direct cell-mineral contact

To determine whether the cyanobacteria had an effect on nutrient release from the regolith, and if so whether that required a direct physical contact between cells and regolith grains, we first incubated 200 kg m^−3^ of MGS-1 in ddH_2_O for 28 days, at 25 °C, before removing regolith grains by centrifugation. We then washed a preculture twice in ddH_2_O and used it to inoculate flasks containing 30 ml of: (i) the water in which regolith had been incubated and from which it had been removed; (ii) ddH_2_O to which 200 kg m^−3^ of MGS-1 had been added on the same day; or (iii) ddH_2_O with 200 kg m^−3^ of MGS-1 contained in a cellulose hydrate dialysis membrane with 15-kDa cutoff (#84568, Reichelt Chemietechnik), selected to prevent direct cell-grain contact but not the diffusion of simple ions and small molecules. Flasks were incubated for 28 days at 25 °C, on a rotary shaker at 100 rpm, under a light intensity of 15–20 μmol_ph_ m^−2^ s^−1^. Once a week, chlorophyll a was extracted with ethanol from the whole of each sample and quantified based on optical density at 665 nm^48^.

### Statistical analysis

Curve fittings and goodness-of-fit tests were performed using GraphPad Prism version 9.2.0 for Windows (GraphPad Software).

### Reporting summary

Further information on research design is available in the Nature Research Reporting Summary linked to this article.

## Supplementary information

Reporting Summary

## Data Availability

The data that support the findings of this study will be made available by the corresponding author upon reasonable request.
